# Exploratory Insights into Gastric Cancer Metabolism Through Amino Acid and Acylcarnitine Profiling in Plasma Samples

**DOI:** 10.3390/biomedicines13092220

**Published:** 2025-09-10

**Authors:** Ștefan Ursu, Cristina-Paula Ursu, Luisa-Gabriela Bogos, Ioana-Ecaterina Pralea, Radu-Cristian Moldovan, Florin Zaharie, Zeno Spârchez, Răzvan Alexandru Ciocan, Rodica Sorina Pop, Cătălin Ioan Bodea, Claudia Diana Gherman, Cristina-Adela Iuga, Nadim Al Hajjar

**Affiliations:** 1 Department of Surgery—Practical Skills, Iuliu Hațieganu University of Medicine and Pharmacy Cluj-Napoca, 400012 Cluj-Napoca, Romania; ursu_stefan@elearn.umfcluj.ro (Ș.U.); pop_cristina_paula@elearn.umfcluj.ro (C.-P.U.); razvan.ciocan@umfcluj.ro (R.A.C.); gherman.claudia@umfcluj.ro (C.D.G.); 2 Surgery Department, Prof. Dr. Octavian Fodor Regional Institute of Gastroenterology and Hepatology Cluj-Napoca, 400162 Cluj-Napoca, Romania; florinzaharie@yahoo.com (F.Z.); bode_cata@yahoo.com (C.I.B.); nadim.alhajjar@umfcluj.ro (N.A.H.); 3 Department of Personalized Medicine and Rare Diseases, MedFuture Institute for Biomedical Research, Iuliu Hațieganu University of Medicine and Pharmacy Cluj-Napoca, 400012 Cluj-Napoca, Romania; bogos.luisa.gabriela@elearn.umfcluj.ro (L.-G.B.); pralea.ioana@umfcluj.ro (I.-E.P.); iugac@umfcluj.ro (C.-A.I.); 4 Drug Analysis Department, Faculty of Pharmacy, Iuliu Hațieganu University of Medicine and Pharmacy Cluj-Napoca, 400012 Cluj-Napoca, Romania; 5 3rd Surgery Department, Iuliu Hațieganu University of Medicine and Pharmacy Cluj-Napoca, 400012 Cluj-Napoca, Romania; 6 3rd Medical Department, Iuliu Hațieganu University of Medicine and Pharmacy Cluj-Napoca, 400012 Cluj-Napoca, Romania; zsparchez@yahoo.co.uk; 7 Community Medicine Department, Iuliu Hațieganu University of Medicine and Pharmacy Cluj-Napoca, 400012 Cluj-Napoca, Romania; drsorinapop@yahoo.com

**Keywords:** stomach neoplasm, metabolomics, amino acids, energy metabolism

## Abstract

**Background**: Gastric cancer ranks fifth among the most prevalent malignancies, with poor prognosis due to limited early-stage diagnosis. Metabolic reprogramming plays a central role in GC development, sustaining carcinogenic processes. **Methods**: In this study, flow-injection tandem mass spectrometry was used to analyse plasma amino acids and acylcarnitines in 62 gastric cancer patients and 70 healthy individuals. Metabolic profiles were correlated with clinical parameters, tumour histology, and recurrence. **Results**: Gastric cancer patients showed significantly reduced levels of Trp, Arg, Tyr, Met, and sum of aromatic AAs—metabolites usually implicated in supporting tumour cell growth and proliferation. At the same time, elevated unsaturated, hydroxylated, and dicarboxylic acylcarnitines suggest mitochondrial and peroxisomal dysfunction. Marked metabolic heterogeneity was observed across histological subtypes, with the indeterminate subtype exhibiting the most pronounced disruption in fatty acid oxidation and widespread acylcarnitine alterations. Decreased levels of C6DC-carnitine and Cit synthesis were correlated with higher tumour recurrence, warranting further confirmatory investigations. **Conclusions**: These findings underscore the value of plasma profiling of amino acids and acylcarnitines for understanding gastric cancer biology, revealing distinct metabolic adaptations reflecting tumour biology, histological subtype, and treatment response.

## 1. Introduction

Gastric cancer (GC), one of the most aggressive malignancies worldwide, ranks fifth in incidence and general cancer-related mortality [1], with poor prognosis despite advances in surgical techniques, chemotherapy protocols, and personalized oncological therapies. Late-stage diagnosis caused by non-specific symptoms, high recurrence rates, and restricted availability of reliable non-invasive biomarkers for early detection and prognosis lead to a 5-year survival rate of less than 30% for advanced stages. Therefore, there is an urgent demand for a better of understanding GC pathogenesis and molecular adaptations of the cancer cells in order to develop new diagnostic tools that can detect GC at an early stage, monitor disease progression, and predict treatment response.

A key starting point in addressing GC complexity lies in its histological classification. Based on the most widely used classification introduced by Laurén in 1965 [2], GC is divided into three major types—intestinal, diffuse and indeterminate—each having characteristic features. Intestinal-type gastric tumours are made of cohesive gland-forming cells and are often associated with environmental risk factors such as *Helicobacter pylori* infection and negative dietary habits. This disease usually goes through a series of mucosal changes, from chronic gastritis and atrophic gastritis to intestinal metaplasia and dysplasia. On the other hand, diffuse-type tumours have dyscohesive, infiltrative growth patterns, often associated with signet-ring cells, and are more strongly linked to genetic factors, such as *CDH1* gene mutations. Clinically, the diffuse type is associated with younger age, female sex, peritoneal dissemination, and a poorer prognosis [3]. The World Health Organization (WHO) classifies GC into four main histological subtypes—tubular adenocarcinoma, papillary adenocarcinoma, mucinous carcinoma, and poorly cohesive carcinoma—each subtype reflecting distinct morphological, clinical, and molecular characteristics [4]. The Laurén and WHO classification systems complement one another: the former provides a practical and epidemiologically meaningful structure, while the WHO one extends morphological detail. Both classification systems remain integral to modern pathology, especially as histology and molecular diagnosis have become increasingly integrated.

Several challenges persist in clinical practice. Current diagnostic methods for gastric cancer, including endoscopy and histopathological examination, are accurate, but invasive, expensive, and not suitable for large-scale screening [5]. Conventional serologic markers such as carcinoembryonic antigen (*CEA*), carbohydrate antigen 19-9 (*CA19-9*), and carbohydrate antigen 72-4 (*CA72-4*) are widely used, but lack adequate sensitivity and specificity for early-stage GC detection [6]. However, there is a notable success story in early GC screening: due to the high prevalence of GC cases in East Asia, Japan developed an effective screening protocol combining image-enhanced endoscopy and abdominal barium radiography, which effectively reduced GC mortality and morbidity rates by increasing early-stage detection and enabling less invasive treatments.

Understanding the underlying biological mechanisms of GC remains crucial for improving disease management. Emerging evidence suggests that metabolic dysregulation has a major role in gastric cancer carcinogenesis, as cells undergo extensive metabolic reprogramming to support rapid proliferation and survival under stressful conditions. Besides the well-known Warburg effect, significant amino acid and lipid metabolism alterations have been noticed [7], with recent studies placing amino acids (AAs) and acylcarnitines (ACs) as key players in cancer metabolism influencing tumour proliferation, immune evasion, and chemoresistance [8,9].

Comprehensive analysis of metabolites in biological samples provides an effective tool to elucidate cancer-specific metabolic alterations and discover novel biomarkers. Compared to conventional genomic and proteomic methods, it has a number of benefits, such as the capacity to identify early metabolic disturbances linked to carcinogenesis and reflect physiological changes in real time [10,11].

Amino acids, apart from their essential role of serving as protein building blocks, are involved in nitrogen metabolism, cellular signalling, and redox balance. Their dysregulation has been reported in GC, including altered levels of Trp, Arg, Met, Gln, Ser, Thr, and others [12,13,14]. Furthermore, altered AC metabolism is associated with mitochondrial dysfunction and disrupted lipid metabolism, both linked to increased proliferation and survival of cancer cells [5], with previous studies identifying elevated levels of adipylcarnitine (C6DC), hexanoylcarnitine (C6), carnitine (C0), and 3-hydroxy-hexadecanoylcarnitine (C16OH) in GC patients as potential novel GC markers [11].

Considering that cancer aggressivity is largely dependent on tumour histology, the literature provides limited data on the metabolic characterisation of GC histological subtypes. A recent genome-scale study highlighted distinct energy production mechanisms in intestinal and diffuse subtypes, the latter relying less on fatty acid oxidation and more on pathways related to glycosaminoglycans [15]. However, these findings have not yet been confirmed at metabolome level.

Considering AAs and ACs as potential targets for diagnostic and therapeutic strategies, this study aimed to provide insights into gastric cancer metabolism through profiling of plasma samples collected from GC patients and healthy controls. Notably, this targeted panel was based on a clinically validated, widely accessible LC-MS/MS assay, routinely used in newborn screening. By applying this established protocol to gastric cancer, this study explored the translational relevance and potential repurposing of a routinely implemented clinical assay in understanding gastric cancer.

## 2. Materials and Methods

### 2.1. Patient Characteristics and Sample Collection

All participants in this study were recruited at the Surgery Department of the Prof. Dr. Octavian Fodor Regional Institute of Gastroenterology and Hepatology in Cluj-Napoca between February 2023 and March 2024. Considering the exploratory aim of this research, a study power of at least 80% was targeted, enabling the detection of medium (Cohen’s *d* = 0.5) to large (*d* = 0.8) effect sizes in a two-sample comparison (GC vs. controls) at *α* = 0.05, resulting in a required sample size of around 64 participants per group. Therefore, the studied cohort included 62 adult patients diagnosed with gastric cancer (TNM stages I–III), 35 of whom had received between 4 and 8 cycles of FLOT neoadjuvant therapy (comprising 5-fluorouracil, leucovorin, oxaliplatin, and docetaxel), the last treatment dose having been administered 4 to 6 weeks prior to sample collection. The control group (CTR) consisted of 70 subjects without gastric malignancies, matched to the gastric cancer patients by age and gender. Demographic and clinicopathological data of the study participants are presented in Table 1.

None of the recruited patients suffered from acute inflammatory diseases, severe uncontrolled diabetes mellitus, severe heart failure (NYHA class IV), chronic kidney disease stage 4 or higher (according to KDOQI classification), or medical history of any other malignancies within the last 5 years. All GC patients underwent follow-up evaluations at 1, 6, and 12 months after hospital discharge. These assessments included routine blood tests, measurement of carcinoembryonic antigen, and abdominal ultrasonography. At 6 months postoperatively—typically following completion of adjuvant chemotherapy—the patients were additionally referred for a CT scan. Disease recurrence was defined in cases with radiological evidence suggestive of relapse (e.g., confirmed tumour mass, distant metastases, extensive peritoneal metastases, or ascites raising suspicion of peritoneal carcinomatosis) and subsequently verified by biopsy or cytological analysis of peritoneal fluid.

Blood samples were collected from GC patients before surgical treatment and processed using a standardized protocol. Blood was drawn by venepuncture in tubes that contained sodium heparin as anticoagulant. Plasma was separated by centrifugation (1300 rcf, 10 min, 4 °C), divided into several aliquots, and stored at −80 °C until analysis. All samples were collected after overnight fasting (last meal typically 7–9 h before sample collection; usual timeframe of sample collection: 6–7 a.m.).

### 2.2. Analysis of Amino Acids and Acylcarnitines

AA and AC analysis was conducted using flow-injection analysis tandem mass spectrometry (FIA-MS/MS) after derivatisation (with 10% acetyl chloride in 1-butanol (*v*/*v*), (Merck, Darmstadt, Germany)), using the same methodology and instrumentation as Avram et al. [16], resulting in a total of 112 metabolites and metabolism indicators. Metabolism indicators are made up of several metabolite sums or ratios that have a biological meaning (e.g., reflect the activity of a certain enzyme). More information on metabolism indicators is provided in Supplementary Information S1. In brief, the sample processing consisted in extracting 5 µL of plasma with methanol (Merck, Darmstadt, Germany) (containing isotope-labelled AAs and ACs (cat. NSK-A and NSK-B, purchased from Cambridge Isotope Laboratories, Tewksbury, MA, USA)). The resulting solution was filtered and then evaporated under a nitrogen stream. The residue was subjected to derivatisation for 20 min at 65 °C. After a second evaporation, the samples were reconstituted in 100 µL of mobile phase prior to FIA-MS/MS analysis.

### 2.3. Data Processing and Statistical Analysis

Raw data were processed using the IonLynx module of MassLynx software (v. 4.2) (Waters Corporation, Milford, MA, USA). The results were exported (Supplementary Table S1) and further statistical analysis performed using MetaboAnalyst 6.0 (available at www.metaboanalyst.ca, accessed on 10 March 2025). The data matrix contained 0.9% missing values, which were estimated using the KNN (feature-wise) option, then the data were log-transformed and mean-centred. Group differences were evaluated using two-sample t-tests using a significance threshold of *p* ≤ 0.05, whereas multiple group comparisons were performed using ANOVA employing the same significance threshold.

IBM SPSS Statistics version 26 (IBM, Armonk, NY, USA) software was utilized to conduct statistical analysis of clinical and paraclinical data. The Shapiro–Wilk test was employed to assess variable distribution. Normally distributed quantitative variables are expressed as arithmetic means ± standard deviation (SD) or percentages, where applicable. In contrast, abnormally distributed variables are reported as medians along with interquartile ranges (25th–75th percentiles). Considering that the cohort sample size was designed to provide 80% power to detect medium-to-large effect sizes (Cohen’s *d* = 0.5–0.8) at the nominal level (*α* = 0.05), the significance threshold for all tests conducted was set at *p* = 0.05. Correlation analysis was performed within SPSS, where either Kendall tau correlation or chi-squared tests were used for the measurement of the strength and direction of association between variables. Cytoscape software [17] (v. 3.9.1, available at https://cytoscape.org) was used for network analysis and visualisation. The MetScape plugin (v. 3.1.3) for Cytoscape was used to construct a network based on correlation analysis using the following parameters: only variables (nodes) with statistically significant correlations (*p* ≤ 0.05) were included for network representation. The minimum considered strength of correlations (edges) was moderate, corresponding to a |*τb*| value of at least 0.26. Examination of the relationship between metabolite levels, recurrence status at 6 months, and treatment was performed using multiple linear regression analysis using GraphPad Prism v. 10.4.1 (GraphPad Software, Boston, MA, USA). For each metabolite, a separate regression model was created, with metabolite concentration (e.g., C6DC, Cit synthesis) as the dependent variable and treatment group (Group: 0 = untreated, 1 = treated) and recurrence at 6 months (R6M: 0 = no recurrence, 1 = recurrence) as independent variables (covariates).

## 3. Results

### 3.1. Correlations Between Clinicopathological Data and Metabolites

**Figure 1:**
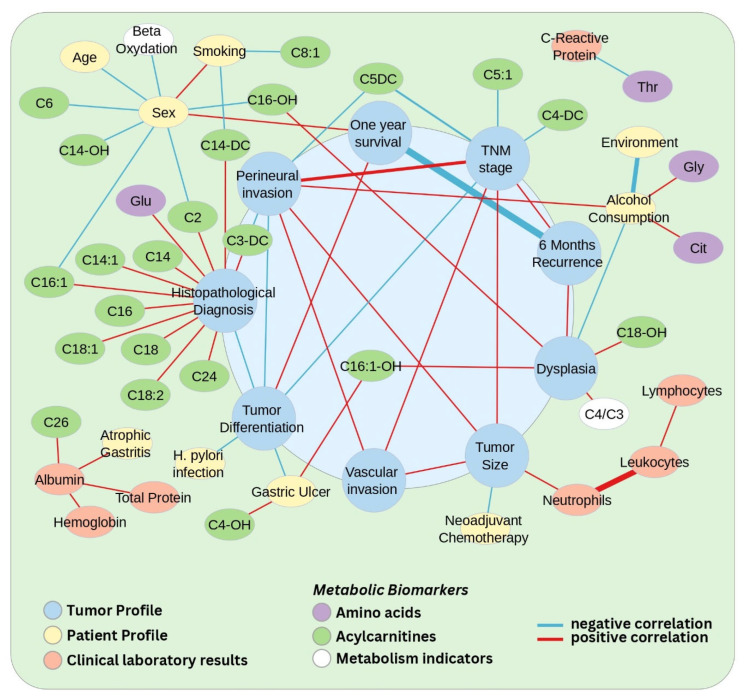
Network representation of significant correlations (raw *p*-value ≤ 0.05, |*τ*| ≥ 0.26) between demographic/clinical characteristics and plasma metabolites in gastric cancer patients. Nodes are color-coded by category: clinical features related to the tumour (blue), patients (yellow), clinical laboratory results (red), and metabolic biomarkers (AAs—violet, ACs—green, metabolism indicators—white). Node size does not carry analytical meaning, and is used only for visualisation of feature category. The edges represent the correlation level between two features, with blue indicating negative correlations and red indicating positive correlations. The width of the edge is proportional to the strength of the correlation. Due to the density of the network, a zoomed and annotated version is provided in Supplementary Figure S1 for improved visualisation (image generated using Cytoscape v3.9.1).

Correlations between clinical features and metabolites in the GC cohort are presented in Figure 1, Supplementary Figure S1, and Supplementary Table S2. These correlations support the general clinical understanding of tumour behaviour, with positive correlations being observed between tumour-specific variables such as tumour size, perineural invasion, vascular invasion, and TNM stage, consistent with the notion that more advanced tumours tend to exhibit more aggressive behaviours, including increased size and enhanced invasion capabilities. Additionally, tumours at higher TNM stages were generally more aggressive and had a higher likelihood of recurrence. Similarly, the negative correlations observed between tumour differentiation and TNM stage or perineural invasion and between tumour size and neoadjuvant chemotherapy further support the understanding that well-differentiated tumours are generally less aggressive. In the GC group, the presence of gastric ulcer or *H. pylori* infection correlated negatively with tumour differentiation, emphasizing the contribution of these two factors to the development of more aggressive, less differentiated tumours. Within the GC group, the 1-year survival variable correlated negatively with 6-month recurrence, emphasizing that recurrence is often associated with poorer survival. Moreover, the positive correlation found between one-year survival and tumour differentiation additionally confirms that well-differentiated tumours are associated with better survival outcomes.

Notably, a positive correlation was observed between perineural invasion (Pn) and patient alcohol consumption. Also, the presence of dysplasia was linked to a higher likelihood of recurrence and was associated with lower alcohol consumption within the GC group. Also, dysplasia was positively correlated with certain long-chain ACs like C18-OH and C16:1-OH and one AC ratio, namely C4/C3. Both Pn and TNM stage showed negative correlations with the levels of short-chain ACs, such as C5-DC, C3-DC, C5:1, and C4-DC.

Patients’ gender and smoking habits showed negative correlations with several ACs, especially long-chain ones. The strong positive correlation between sex and smoking indicates that smoking behaviours differ between sexes in this cohort. Additionally, sex appears to have a notable influence on C2, C6, and the long-chain ACs C16:1, C14:OH, and C16-OH.

The correlation analysis confirmed well-established physiological relationships, particularly between albumin and total protein, as well as haemoglobin levels. Additionally, the correlation between leucocyte, neutrophil, and lymphocyte counts was in line with expected immune system dynamics. An intriguing finding was the negative correlation between C-reactive protein (CRP) and threonine levels. Furthermore, the positive correlation between neutrophil count and tumour size was particularly noteworthy, supporting the idea that tumour-associated inflammation may influence tumour growth.

### 3.2. Metabolic Alterations Induced by Gastric Cancer

**Figure 2:**
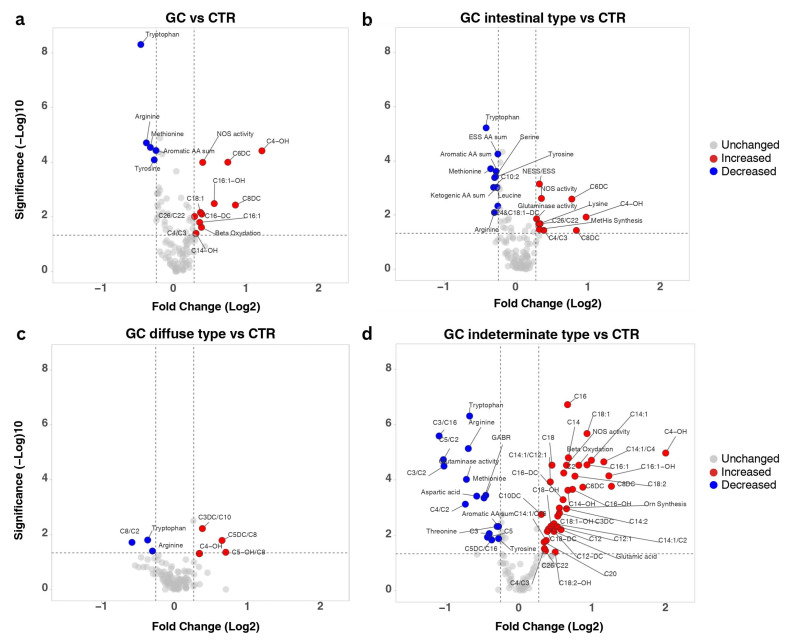
Volcano plot depicting significantly modified metabolites (raw *p*-value ≤ 0.05, |*FC*| ≥ 1.2) (**a**). Gastric cancer group (n = 62) compared to controls (n = 70). (**b**–**d**) Comparisons between the three different histological types (**b**—intestinal (n = 34), **c**—diffuse (n = 13), **d**—indeterminate (n = 15)) compared to controls (n = 70). Significantly altered metabolites are shown in red (increased) and blue (decreased), while metabolites that did not show any significant change are represented in grey. In each plot, the horizontal dotted line represents a significance threshold of a raw *p*-value ≤ 0.05, while the vertical ones represent the thresholds of |*FC*| ≥ 1.2 (image generated using the VolcaNoseR web app, available at https://goedhart.shinyapps.io/VolcaNoseR/, accessed on 26 March 2025).

AA and AC profiling of plasma samples collected from gastric cancer patients revealed 48 significantly altered metabolites (Supplementary Table S3) compared to the CTR, with 17 metabolites showing a |*FC*| > 1.2 (Figure 2a). Among the altered AAs, Trp, Arg, Tyr, Met, and the sum of aromatic AAs presented decreased concentrations in the GC patients’ samples. In contrast, increases were observed for unsaturated, hydroxylated, and dicarboxylic ACs such as C4-OH, C6DC, C8DC, C16DC, C16:1, and C18:1. Additionally, increases in metabolism indicators were observed in the GC group, particularly for NOS activity, as indicated by the Cit/Arg ratio, and in beta oxidation, as reflected by (C2 + C3)/C0 ratio.

The impact of tumour histology (according to Laurén classification [2]) on metabolism can be observed in Figure 2b–d as significantly modified metabolites (*p* ≤ 0.05, |*FC*| > 1.2). The number of significantly modified metabolites greatly differed between the three subtypes of GC, intestinal, diffuse, and indeterminate (Supplementary Tables S4–S6), with only three shared changes compared to CTR: decreased levels of Trp and Arg and increased C4-OH. Except these, intestinal GC was characterized by generally down-regulated amino acid metabolism, with decreased Trp, Met, Tyr, Arg, Ser, and Leu and diminished levels of essential and aromatic AAs. Notably, Lys levels were increased along with glutaminase activity and methylhistidine synthesis. The highest quantitative increases in intestinal GC were observed for three ACs—C4-OH, C6DC, and C8DC. In contrast, the diffuse type of GC exhibited modifications primarily in C8-carnitine related ratios (increased C3DC/C8, C5DC/C8, and C5-OH/C8, decreased C8/C2). The highest number of significantly altered metabolites was observed for the indeterminate type of GC. All measured long-chain ACs and half the medium-chain ones were elevated, as well as C20, one of the very-long-chain ACs. Decreased concentrations were determined for two short-chain ACs (C3 and C5), whereas C2, C3DC, C4-OH, and C6DC were increased. Imbalances in fatty acid oxidation was also observed through several altered AC ratios (decreased: C3/C16, C5/C2, C3/C2, C4/C2, C5DC/C16; increased: C4/C3, C14:1/C12:1, C14:1/C2, C26/C22). Except Trp and Arg, which were decreased across all histological subtypes, the indeterminate type had reduced levels of aromatic AAs, Asp, Met, Tyr, Thr, glutaminase activity, and GABR. Concurrently, Glu, Orn synthesis, and NOS activity were significantly up-regulated. Given that all histological subtypes were compared to the same control group, the metabolites uniquely altered in each subtype may represent discriminative metabolic signatures. Also, several metabolites showed subtype-specific trends. For instance, both C4-OH and C8DC exhibited the highest fold changes in the indeterminate subtype, moderate increases in the intestinal subtype, and the lowest levels in the diffuse subtype. In contrast, glutaminase activity showed divergent trends in the indeterminate and intestinal subtypes.

Chemotherapy usually leads to modifications in metabolite levels. To ensure that the observations made in the previous comparisons were induced by GC, the impact of FLOT chemotherapy within the GC group was investigated, even though sampling of treated patients was performed after 4-6 weeks after the last treatment dose.

**Figure 3:**
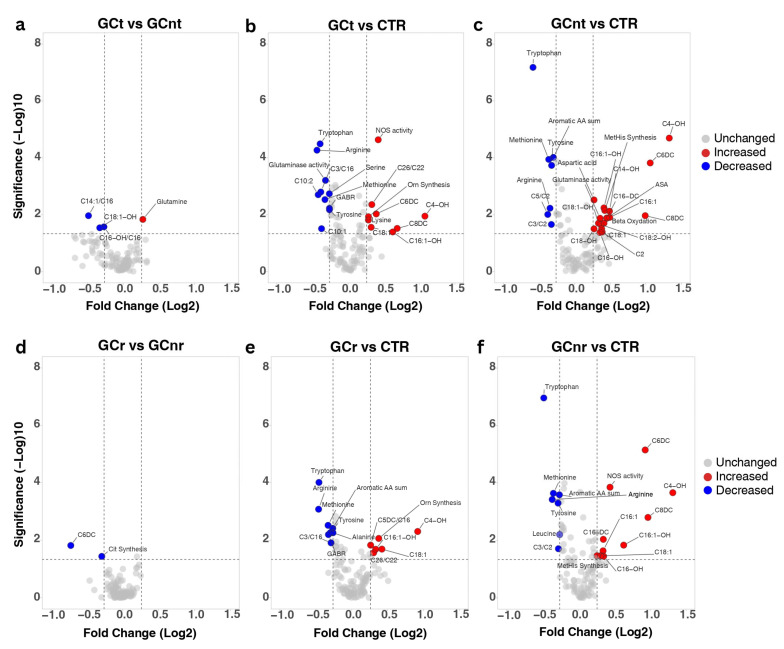
Volcano plots depicting the significantly modified metabolites (raw *p*-value ≤ 0.05, |*FC*| ≥ 1.2) related to (i) neoadjuvant chemotherapy, when comparing: (**a**) treated (n = 35) and untreated (n = 27) patient groups; (**b**) treated (n = 35) and control (n = 70) groups; (**c**) untreated (n = 27) and control (n = 70) groups; (ii) 6-month recurrence of gastric cancer, when comparing: (**d**) patient groups with recurring (n = 21) and non-recurring (n = 41) gastric cancer; (**e**) recurring gastric cancer group (n = 21) and healthy controls (n = 70); (**f**) non-recurring gastric cancer group (n = 41) and healthy controls (n = 70). Significantly altered metabolites are shown in red (increased) and blue (decreased), while metabolites that did not show any significant change are represented in grey. In each plot, the horizontal dotted line represents a significance threshold of a raw *p*-value ≤ 0.05, while the vertical ones represent the thresholds of |*FC*| ≥ 1.2 (image generated using the VolcaNoseR web app, available at https://goedhart.shinyapps.io/VolcaNoseR/, accessed on 26 March 2025).

Five significantly altered metabolites and metabolite ratios were found when comparing the GC patients that received neoadjuvant chemotherapy (GCt) to the ones that did not receive any treatment (GCnt) prior to surgery (Supplementary Table S7). Notable increases were observed for Gln, whereas C4-carnitine, long-chain hydroxylated AC C18:1-OH, and two AC ratios (C14:1/C16 and C16-OH/C16) presented decreased levels (Figure 3a). To better understand the residual effect of FLOT chemotherapy, a sensitivity analysis excluding the treated patients was conducted by comparing each metabolite’s effect sizes (Cohen’s d). Effect directions and percentage changes are summarized in Supplementary Table S8. Decreased levels of Trp, Met, Tyr, and aromatic AA sum were reinforced by sensitivity analysis (changes in effect size of Trp −35%, Met −9%, Tyr −16%, aromatic AA sum −17%). At the same time, the observed significant increases in the GC group were further supported by changes in effect size for C4-OH (+51%), C6DC (+44%), C8DC (+28%), C16:1-OH (+14%), C14-OH (+103%), C16DC (+22.5), C16:1 (+52%), C18:1 (+29%), C4/C3 (+39.4%), and beta oxidation (+29.2%). For Arg, the treatment subgroup may have amplified the deficit (+9%), but it remained significantly decreased compared to controls, while for NOS activity the effect weakened and lost significance once treated patients were excluded. At the same time, several metabolites were found to be masked by treatment (effect became stronger after exclusion): MetHis synthesis, ASA, C2, C16-OH, C18-OH, C18:1-OH, and C18:2-OH (all shifted to medium positive effects in sensitivity). When compared to the CTR group, the two gastric cancer subgroups (with and without treatment) exhibited several differences in terms of metabolite significance (Figure 3b,c, Supplementary Tables S9 and S10). Nine metabolites and metabolism indicators were modified exclusively in the GCt vs. CTR comparison, with NOS activity being the most significant variable. In contrast, for GCnt vs. CTR, NOS activity did not meet the selected significance threshold. Sixteen significant metabolites were exclusively recorded in the GCnt group when compared to the CTR group, with most of these metabolites belonging to long-chain, hydroxylated, or unsaturated ACs (C14-OH, C16:1, C16-DC, C16-OH, C18-OH, C18:1-OH, and C18:2-OH). Ten metabolites were commonly found in both GCnt and GCt when compared with CTR, including several AAs such as Arg, Met, Trp, Tyr, medium-chain ACs C4-OH, C6DC, and C8DC, and long-chain C16:1-OH, and C18:1. Among these, C6DC, C4-OH exhibited the greatest difference in terms of fold change between the GC groups and CTR, generally showing higher fold changes in the GCnt group. In particular, glutaminase activity was reduced in GCt group, but elevated in the untreated GC group relative to CTR.

### 3.3. Metabolites Linked to GC Recurrence

Two features, namely C6DC and Cit synthesis, were identified as significantly altered (*p* ≤ 0.05, |*FC*| > 1.2—see Supplementary Table S11) and linked to GC recurrence (Figure 3d). For C6DC, regression analysis revealed significant independent effects of both recurrence (β = −0.01970, *p*-value: 0.0487) and treatment group (β = −0.01745, *p*-value: 0.0196) on metabolite concentrations, with no significant interaction between these factors (β = 0.01052, *p*-value: 0.4113). C6DC levels were consistently reduced in patients with recurrence regardless of treatment (R^2^ = 0.1582), indicating independent effects.

In contrast, Cit synthesis displayed a significant interaction between recurrence and treatment (β = 0.02917, *p*-value: 0.0136), with recurrence alone being a strong negative predictor of cit Synthesis (β = −0.02988, *p*-value: 0.0013), while treatment alone had no significant effect (β = −0.008054, *p*-value: 0.2240) on Cit synthesis. This interaction indicates that Cit synthesis reduction is associated with recurrence only in untreated patients. These findings show that Cit synthesis may be a treatment-responsive marker of recurrence, while decreased levels of C6DC reflect a more general signature of recurrence regardless of treatment.

Additional insights were obtained by comparing the recurring (GCr) and non-recurring (GCnr) groups with the healthy controls (Figure 3e,f, Supplementary Tables S12 and S13). Six metabolites and indicators were uniquely altered in the GCr group, namely Ala, GABR, C3/C16, C5DC/C16, C26/C22, and Orn synthesis. In contrast, the non-recurring group was specifically distinguished from the CTR by alterations in Leu, C6DC, C8DC, C16:1, C16-OH, and C3/C2, together with MetHis synthesis and NOS activity. Besides Trp, Arg, Met, Tyr, and the sum of aromatic AAs, three ACs were commonly reported as differentiating both the GCr and GCnr groups from CTR: C4-OH, C18:1, and C16:1-OH. Generally, these common metabolites showed a higher fold change and significance in differentiating the GCnr group from CTR, whereas increased levels of C6DC, C8DC, and NOS activity could be linked with absence of 6-month recurrence.

## 4. Discussion

Profiling of AAs and ACs is a widely available assay commonly used for detecting inborn errors of metabolism during newborn screening. Considering the implications of these classes of metabolites in multiple metabolic pathways, there have been attempts to evaluate their roles in other diseases such as heart conditions [18], metabolic disorders [19,20], and cancer [16,21], including gastric cancer [11]. This study was designed to evaluate the metabolic implications of AAs and ACs in gastric cancer pathogenesis, highlighting valuable insights regarding possible roles of these metabolites in predicting tumour recurrence or evaluating therapeutic efficacy.

Correlation analysis of tumour clinical variables aligned with established tumour behaviour patterns (e.g., the negative correlation of tumour differentiation with TNM stage and perineural invasion, neoadjuvant chemotherapy negatively correlated with tumour size, etc.). Chronic pathological states such as atrophic gastritis (67.7%), gastric ulcers (48.4%), and *H. pylori* infection (51.6%) were highly prevalent among GC patients. Both *H. pylori* infection and gastric ulcers negatively correlated with tumour differentiation, suggesting a potential relationship between these two factors and GC aggressiveness. On the other hand, the presence of atrophic gastritis was correlated only with physiological markers such as albumin and total protein levels measured preoperatively. These conditions are well-established risk factors contributing to gastric carcinogenesis, highlighting their potential role in disease development. In terms of lifestyle habits, 51.6% of GC patients were smokers and 24.2% reported regular alcohol consumption. Within this cohort, alcohol consumption was positively correlated with perineural invasion capabilities of the tumour. While both variables are known correlates of tumour aggressiveness [22,23], the direct link between them has not yet been studied in the context of gastric cancer.

Clinically, a substantial proportion of patients presented with advanced disease, with 53.2% at TNM stage III. Histopathological evaluation revealed that tubular adenocarcinoma was the predominant subtype, accounting for 54.8% of cases (corresponding to the Laurén intestinal type), followed by adenocarcinoma with mixed subtypes (24.2%—Laurén GC diffuse type), poorly cohesive carcinoma (11.3%), and signet ring cell carcinoma (9.7%) (both indeterminate subtypes according to Laurén classification). Additionally, nearly half the GC patients (43.5%) exhibited poorly differentiated tumours, which generally correlate with aggressive disease progression and worse clinical outcomes.

All GC patients underwent surgical intervention as the primary treatment, while neoadjuvant chemotherapy (FLOT) was administered to 56.5% of patients. Despite these interventions, recurrence of the disease occurred in 33.9% of the patients within six months post-surgery, highlighting an ongoing challenge in the management of gastric cancer and emphasizing the need for enhanced diagnostic accuracy and therapeutic approaches to improve patient outcomes.

Fatty acid oxidation is one of the main mechanisms for energy production in order to support different cellular activities. Carnitine plays a distinct role by conjugating with fatty acids, facilitating their transport from cytosol into the mitochondrial matrix as ACs. Dysregulated AC levels have been increasingly associated with digestive cancers, such as hepatocellular cancer [24], colorectal cancer [16,25], or gastric cancer [11]. The roles of ACs in health and disease are comprehensively discussed in a review paper by Dambrova et al. [26].

Compared to the control group, the metabolites with the most elevated concentrations in GC patients were ACs, AC-related metabolism indicators, such as beta oxidation, and one AC ratio—C24/C22. Increased fatty acid metabolism expressed through elevated beta oxidation reflects the high energy demands induced by the tumoral tissue. Moreover, elevated expression of some unsaturated long-chain (C16:1, C18:1), dicarboxylic (C16-DC), and hydroxylated (C16:1-OH) ACs have previously been linked to alterations in mitochondrial beta oxidation [26], whereas very-long-chain AC imbalance, such as the one expressed through the C24/C22 ratio, usually reflects peroxisomal disorders [27,28].

Based on tumour histology, indeterminate GC was characterized by a very high number of up-regulated ACs, including all long-chain ACs, together with most of the medium-chain ones (Figure 2d), revealing a very altered state of fatty acid metabolism. This nonspecific accumulation of ACs seems to reflect a significant impairment in the production of energy through fatty acid oxidation, but these observations require further experimental validation on a cohort of GC patients with indeterminate GC histological subtype. In contrast, fatty acid oxidation seemed to be much less affected in intestinal and diffuse subtypes, confirming previous genome-based reports on metabolic dysregulation of energy metabolism [29].

Among all measured metabolites, the highest quantitative variation compared to healthy subjects was recorded for 3-hydroxybutyrylcarnitine (C4-OH). This AC is formed from 3-hydroxybutyric acid (3HB), a ketone body usually associated with fasting and ketosis. Considering the gastric pathology, this observation was expected. Nevertheless, 3HB has been previously documented to be both a signalling molecule [30] and an energy source. Its use by GC cells as an energy source was highlighted by Zhou et al. [31], who documented decreased levels of C4-OH in tumoral tissue compared to adjacent tissue. Moreover, increased concentrations of 3HB can modulate gene expression by inhibiting histone deacetylase [32] and suppressing the NLRP3 inflammasome [33].

Three dicarboxylic ACs (C6DC, C8DC, and C16-DC) were increased in plasma of GC patients. These ACs can be synthesized by peroxisomes [34], their increase supporting the hypothesis of peroxisomal dysfunction present in the malignant tissue. Of particular interest are the two up-regulated medium-chain dicarboxylic ACs adipylcarnitine (C6DC) and suberylcarnitine (C8DC), which seem to be linked to gastro-intestinal malignancies, having also been previously documented as significantly upregulated in gastric [11] and colorectal [16] cancers, both studies underlining the usefulness of C6DC either for differentiating between GC, atrophic gastritis, and superficial gastritis or proposed as diagnostic biomarkers for colorectal cancer. Moreover, these two metabolites were found to be non-discriminatory between GC patients with tumour recurrence and the healthy control group, but only C6DC managed to discriminate between patients with tumour recurrence and those without. Moreover, this significant decrease in levels of C6DC recorded in the GCr group was not influenced by the neoadjuvant treatment received by the patients, and therefore normal concentrations recorded for GC patients might have predictive value for tumour recurrence, a possibility worth further investigation.

Two hydroxylated long-chain ACs (C14-OH and C16:1-OH) were significantly increased in the GC group, which indicates decreased activity of enoyl coenzyme A hydratase short-chain 1 (ECHS1), an enzyme implicated in mitochondrial β-oxidation [35]. Moreover, the patients that did not receive neoadjuvant chemotherapy showed multiple elevated long-chain hydroxy ACs (C14-OH, C16-OH, C16:1-OH, C18-OH, C18:1-OH, C18:2-OH) compared to the healthy controls. Increased concentrations of long-chain fatty acids were observed in cardiac tissue during myocardial ischemia, caused by blocked mitochondrial fatty acid oxidation [36,37]. A similar mechanism might also be involved in GC, considering that the tumours are characterized by hypoxia, a key factor in tumour progression, invasion, and metastasis formation [38].

Metabolic reprograming of AAs observed for the GC patients was exhibited through significantly decreased levels of Trp, Arg, Met, Tyr, and the sum of aromatic AAs. Alterations in the metabolism of Trp and Arg have been associated with different digestive cancers [39,40,41], whereas Met, an essential amino acid, contributes to different areas of cell metabolism, such as methylation reactions, polyamine synthesis, or redox maintenance [42]. At the same time, decreased levels of the sum of aromatic AAs (comprising Trp, Tyr and Phe) can have a pro-proliferative effect, considering that recent evidence points out these three AAs modulate proteasome translocation via the mTOR pathway, modulation that is mediated through sestrin 3 [43,44]. Cancer cell growth can also be supported by increased demand of essential AAs [45], as observed for the intestinal GC histological subtype, together with decreased levels of ketogenic AAs, which can be directly metabolised to acetyl-CoA to ensure the necessary energy demands.

The implications of Trp metabolism in digestive malignancies have been extensively studied [10,39,46,47,48]. Trp degradation is considered an adaptation of the tumour cell against acute immune response [10,47]. In the tumour microenvironment, Trp depletion is mainly achieved by its degradation to kynurenine by overexpressed indoleamine 2,3-dioxygenase (IDO) [49]. IDO is implicated in controlling local adaptive immunity by augmenting inflammation and suppressing immunological responses of cytotoxic T cells, its expression being modulated by several Trp metabolites [50,51]. IDO’s expression was found to be increased [52] in the gastric mucosa of *H. pylori*-infected individuals, and contributes to modulation of the Th1/Th2 and Th17 pathways. Nevertheless, decreased levels of Trp have also been connected with microbiome imbalances and inflammation [50].

The significantly modified metabolism of Arg is in accordance with previous reports of GC-associated metabolic alterations [40,53]. Arg is implicated in several cellular processes, such as the synthesis of nitric oxide, a molecule that plays complex roles in tumour biology ranging from cytotoxic effects in immune surveillance to pro-tumorigenic functions in hypoxic conditions [40,46]. Patients that did not receive neoadjuvant chemotherapy prior to sampling also exhibited increased levels of argininosuccinic acid, which is an intermediary product in Arg synthesis from Cit mediated by argininosuccinate synthetase 1 (ASS1). Increased expression of ASS1 has been observed in GC, and is closely linked to the migration capacity of GC cells [54].

Upregulated synthesis of Orn was recorded for patients who had tumour recurrence (Figure 3e) concurrent with decreased Cit synthesis compared to the non-recurring group (Figure 3d). These findings suggest that alterations in the arginine pathway are involved in the tumour recurrence mechanism and may serve as potential leads towards clinically addressing disease severity and recurrence [55]. Increased Orn synthesis was also recorded for the indeterminate histological subtype, while enhanced ornithine metabolism has previously been correlated with aggressive cancer phenotypes by supporting rapid tumour proliferation and cellular growth [40].

The significantly lower levels of Met recorded for the GC group are in accordance with previous reports on the dependence of GC on Met, evidence suggesting that Met has an important role in the progression of GC [56,57]. Met is involved in methylation of nucleic acids and proteins, synthesis of polyamines, or redox maintenance [42]. A central source of methyl groups, Met can modulate autophagy, apoptosis, and ferroptosis in GC cells [58]. Overexpression of γMETase caused by Met depletion promotes autophagy and apoptosis of GC cells [59], suggesting that decreased levels of Met may stimulate the proliferation of GC cells.

Systemic anti-cancer medication can induce marked metabolic alterations [60]. Considering that the GC patients that received FLOT chemotherapy were sampled after 4-6 weeks after the last chemotherapy dose, they exhibited limited changes compared to the group of patients that did not receive the treatment. The sensitivity analysis conducted after eliminating the GC patients that received FLOT chemotherapy strengthened most of the observations that were made on the entire cohort, highlighting limited residual effects of the treatment, except for NOS activity, the effect size of which seemed to be induced by the treated subgroup. The small, but significant increase in Gln seen after treatment seems to be primarily related to a marked decrease in glutaminase activity. At the same time, down-regulation of certain ACs (C4 and C18:1-OH) and AC ratios (C14:1/C16, C16-OH/C16) indicates limited impact of FLOT chemotherapy on mitochondrial function.

The main limitation of this study comes from the relatively small number of observations. As this study was exploratory in nature, focusing on hypothesis generation rather than definitive biomarker validation, the sample size limitations are obvious, especially when observing metabolic alterations in different GC subgroups, thus making it difficult to further test the specificity of certain findings, such as AC’s alterations dependent on tumour histology. Moreover, application of multiple testing correction inevitably reduces statistical power, particularly for moderate effect sizes. On top of this, the relatively modest sample size, in the context of measuring 112 metabolites, increases the risk of false positives and potential overfitting, even after correction. These issues are inherent to exploratory metabolomic studies and highlight the need for replication and validation in larger independent cohorts.

Another limitation of this study is the lack of information on certain patient-level factors that may influence metabolite profiles, including dietary habits, fasting status, and medication use (other than chemotherapy), as these variables were not systematically recorded in the original patient database. Additionally, patients with GC may have disease-induced changes in diet, fasting behaviour, or medication use, which could further influence metabolite levels. Future studies with prospective data collection should aim to control for these potential confounders to better isolate disease-specific metabolic signatures.

Considering the translational potential of certain findings of this study (such as tumour histology dependent metabolic alterations or recurrence-related metabolites), future efforts should be focused on validating these observations on larger cohorts for better understanding the metabolic differences among different subgroups of GC patients.

Integrating genomics, transcriptomics, proteomics, epigenomics, metabolomics, and microbiomics can illuminate early disease trajectories and progression risks, yielding biomarker panels that outperform single-modality readouts, such as the multi-omic approach for Barrett’s oesophagus and oesophageal adenocarcinoma [61]. Such strategies provide a strong conceptual framework for gastric cancer research, suggesting that multi-layered molecular profiling may overcome the limitations of single-modality biomarkers and better account for the biological heterogeneity across histological subtypes. Multi-omics could be integrated with new technologies, such as fluorescence molecular endoscopy and confocal laser endomicroscopy, that can visualize biomarker-defined lesions in vivo. These methods hold promise for improving biopsy targeting, mapping spatial heterogeneity, and monitoring therapeutic response in real time [62].

## 5. Conclusions

This study aimed to evaluate which metabolic adaptations of GC can be characterized through AA and AC profiling, in order to identify distinct metabolic signatures that could further translate into improved patient care.

Energy metabolism was found to be clearly altered in GC. Alterations in fatty acid oxidation were reflected both by altered mitochondrial beta oxidation (shown by increased levels of several ACs and AC ratios) and by defects in peroxisomal activity (observed through imbalances of very-long-chain ACs and elevated dicarboxylic ACs). Moreover, the AC profile was highly dependent on tumour histology, a major disruption in fatty acid oxidation being observed for the indeterminate GC histological subtype, with almost all ACs significantly modified. In striking contrast, mitochondrial activity was much less affected in diffuse and intestinal GC subtypes. This increased metabolic heterogeneity observed among GC histological subtypes highlights the need for better understanding the underlying pathological mechanisms in order to adapt the therapeutic approach.

AA analysis revealed a series of significantly altered metabolites, characterizing the ways in which GC evades the acute immune response (↓Trp), sustains malignant proliferation (↓Met, ↓Aromatic AAs) and migration (↓Arg), enhances metastatic capability (↑Orn synthesis), or is associated with aggressive cancer phenotypes.

As this was an exploratory metabolomic analysis, multiple comparisons were performed across 112 metabolites. While both nominal *p*-values and FDR-adjusted *q*-values (supplementary tables) are provided, the risk of false positives remains, particularly for subgroup analyses involving smaller numbers of patients (e.g., chemotherapy response, GC type, or recurrence). Consequently, these findings should be considered hypothesis-generating and require validation in independent cohorts.

AA and AC profiling reflects a broad spectrum of metabolic adaptations characteristic of or associated with gastric cancer. Therefore, this approach could prove a useful tool for patient phenotyping in personalized healthcare.

## Figures and Tables

**Table 1:** Demographic and clinicopathological data of the patients and controls.

		GC Patients	CTR
Number		62	70
Sex (% males)		67.7	68.6
Median age (years)		70	70
Percentiles—25		57.8	57.0
Percentiles—75		74.0	74.0
Associated pathologies			
Gastric ulcer		30 (48.4%)	-
Atrophic gastritis		42 (67.7%)	-
*Helicobacter pylori* infection		32 (51.6%)	-
Lifestyle habits			
Smoking		35 (51.6%)	-
Alcohol consumption		15 (24.2%)	-
GC TNM stages			
I		13 (21.0%)	-
II		16 (25.8%)	-
III		33 (53.2%)	-
Tumour histology			
Laurén classification	WHO classification		
Intestinal type	Tubular adenocarcinoma	34 (54.8%)	-
Diffuse type	Signet ring cell carcinoma	6 (9.7%)	-
	Poorly cohesive carcinoma	7 (11.3%)	-
Indeterminate type	Adenocarcinoma with mixed subtypes	15 (24.2%)	-
GC differentiation			
Poorly differentiated		27 (43.5%)	-
Moderately differentiated		21 (33.9%)	-
Well differentiated		14 (22.6%)	-
Treatment			
Neoadjuvant chemotherapy		35 (56.5%)	-
Surgery		62 (100%)	-
6-month tumour recurrence		21 (33.9%)	-
One-year survival		50 (80.6%)	

## Data Availability

Data are provided within the Supplementary Information Files.

## Supplementary Materials

The following supporting information can be downloaded at https://www.mdpi.com/article/10.3390/biomedicines13092220/s1, Supplementary Information S1—List of measured metabolites and metabolite sums and ratios; Supplementary Figure S1: Network representation of significant correlations; Supplementary Table S1: Metabolite measurements; Table S2: Correlation analysis; Table S3: Significantly modified metabolites in gastric cancer vs. controls; Table S4: Significantly modified metabolites in intestinal type gastric cancer vs. controls; Table S5: Significantly modified metabolites in diffuse type gastric cancer vs. controls; Table S6: Significantly modified metabolites in indeterminate type gastric cancer vs. controls; Table S7: Significantly modified metabolites induced by chemotherapy; Table S8:Sensitivity analysis regarding impact on treatment on GC specific metabolites; Table S9: Significantly modified metabolites in untreated gastric cancer vs. controls; Table S10: Significantly modified metabolites in treated gastric cancer vs. controls; Table S11: Metabolites linked to 6-month GC recurrence; Table S12: Significantly modified metabolites in recurring gastric cancer vs. controls; Table S13: Significantly modified metabolites in non-recurring gastric cancer vs. controls.
