# Supplementary material for: Exploratory Insights into Gastric Cancer Metabolism Through Amino Acid and Acylcarnitine Profiling in Plasma Samples
Source: Biomedicines. 2025 Sep 10;13(9):2220. doi: 10.3390/biomedicines13092220 (PMC12466941; doi:10.3390/biomedicines13092220)
Supplement: Supplementary file 1 [file biomedicines-13-02220-s001.zip › AAAC_GC_Supplementary Infomation S1.pdf]

biomedicines

MDPI

Supplementary Information

Exploratory Insights into Gastric Cancer Metabolism Through Amino Acid and Acylcarnitine Profiling in Plasma Samples

Ștefan Ursu [1,2], Cristina-Paula Ursu [1,2], Luisa-Gabriela Bogos [3,4], Ioana-Ecaterina Pralea [3], Radu-Cristian Moldovan [3,*], Florin Zaharie [2,5], Zeno Spârchez [6], Răzvan Alexandru Ciocan [1], Rodica Sorina Pop [7], Cătălin Ioan Bodea [2], Claudia Diana Gherman [1], Cristina-Adela Iuga [3,4] and Nadim Al Hajjar [2,5]

[1] Department of Surgery—Practical Skills, Iuliu Hațieganu University of Medicine and Pharmacy Cluj-Napoca, 400012 Cluj-Napoca, Romania; ursu_stefan@elearn.umfcluj.ro (Ș.U.); pop_cristina_paula@elearn.umfcluj.ro (C.-P.U.); razvan.ciocan@umfcluj.ro (R.A.C.); gherman.claudia@umfcluj.ro (C.D.G.)
[2] Surgery Department, Prof. Dr. Octavian Fodor Regional Institute of Gastroenterology and Hepatology Cluj-Napoca, 400162 Cluj-Napoca, Romania; florinzaharie@yahoo.com (F.Z.); bode_cata@yahoo.com (C.I.B.); nadim.alhajjar@umfcluj.ro (N.A.-H.)
[3] Department of Personalized Medicine and Rare Diseases, MedFuture Institute for Biomedical Research, Iuliu Hațieganu University of Medicine and Pharmacy Cluj-Napoca, 400012 Cluj-Napoca, Romania; bogos.luisa.gabriela@elearn.umfcluj.ro (L.-G.B.); pralea.ioana@umfcluj.ro (I.-E.P.); iugac@umfcluj.ro (C.-A.I.)
[4] Drug Analysis Department, Faculty of Pharmacy, Iuliu Hațieganu University of Medicine and Pharmacy Cluj-Napoca, 400012 Cluj-Napoca, Romania
[5] 3rd Surgery Department, Iuliu Hațieganu University of Medicine and Pharmacy Cluj-Napoca, 400012 Cluj-Napoca, Romania
[6] 3rd Medical Department, Iuliu Hațieganu University of Medicine and Pharmacy Cluj-Napoca, 400012 Cluj-Napoca, Romania; zsparchez@yahoo.co.uk
[7] Community Medicine Department, Iuliu Hațieganu University of Medicine and Pharmacy Cluj-Napoca, 400012 Cluj-Napoca, Romania; drsorinapop@yahoo.com
* Correspondence: moldovan.radu@umfcluj.ro

Supplementary Information S1 – List of measured metabolites and metabolite sums and ratios

	**Metabolite**	**Observations**
1.	Glycine	
2.	Leu&Ile	Sum of Leu and Ile
3.	Ornithine	
4.	Methionine	
5.	Citrulline	
6.	Arginine	
7.	Argininosuccinic acid	
8.	Alanine	
9.	Valine	
10.	Histidine	
11.	Methylhistidine	Sum of 1- and 3-methylhistidine
12.	Proline	
13.	Glutamine	
14.	Phenylalanine	
15.	Lysine	
16.	Serine	
17.	Threonine	
18.	Tyrosine	
19.	Tryptophan	
20.	Aspartic acid	
21.	Glutamic acid	
22.	C0-Carnitine	Free carnitine
23.	C2-Carnitine	Acetylcarnitine

	Metabolite	Observations
24.	C3-Carnitine	Propionylcarnitine
25.	C3DC-Carnitine	Malonylcarnitine
26.	C4-Carnitine	Butyrylcarnitine
27.	C4-OH-Carnitine	3-Hydroxybutyrylcarnitine
28.	C4DC-Carnitine	Succinylcarnitine
29.	C5-Carnitine	Valerylcarnitine
30.	C5:1-Carnitine	Methylcrotonylcarnitine/Tiglylcarnitine
31.	C5-OH-Carnitine	3-Hydroxyvalerylcarnitine
32.	C5DC-Carnitine	Glutarylcarnitine
33.	C6-Carnitine	Caproylcarnitine
34.	C6DC-Carnitine	Adipylcarnitine
35.	C8-Carnitine	Octanoylcarnitine
36.	C8:1-Carnitine	Octenoylcarnitine
37.	C8DC-Carnitine	Suberylcarnitine
38.	C10-Carnitine	Decanoylcarnitine
39.	C10:1-Carnitine	Decenoylcarnitine
40.	C10:2-Carnitine	Decadienoylcarnitine
41.	C10DC-Carnitine	Decanedioylcarnitine
42.	C12-Carnitine	Lauroylcarnitine
43.	C12:1-Carnitine	Dodecenoylcarnitine
44.	C12-DC-Carnitine	Dodecanedioylcarnitine
45.	C14-Carnitine	Myristoylcarnitine
46.	C14:1-Carnitine	Tetradecenoylcarnitine
47.	C14:2-Carnitine	Tetradecadienoylcarnitine
48.	C14-OH-Carnitine	3-Hydroxy-tetradecanoylcarnitine
49.	C14-DC-Carnitine	Tetracanedioylcarnitine
50.	C16-Carnitine	Palmitoylcarnitine
51.	C16:1-Carnitine	Hexadecenoylcarnitine
52.	C16-OH-Carnitine	3-Hydroxy-hexadecanoylcarnitine
53.	C16:1-OH-Carnitine	3-Hydroxy-hexadecenoylcarnitine
54.	C16-DC-Carnitine	Hexadecanedioylcarnitine
55.	C18-Carnitine	Stearoylcarnitine
56.	C18:1-Carnitine	Octadecenoylcarnitine
57.	C18:2-Carnitine	Octadecadienylcarnitine
58.	C18-OH-Carnitine	3-Hydroxysteroylcarnitine
59.	C18:1-OH-Carnitine	3-Hydroxyoleylcarnitine
60.	C18:2-OH-Carnitine	3-Hydroxylinoleoylcarnitine
61.	C18-DC-Carnitine	Octadecanedioylcarnitine
62.	C20-Carnitine	Eicosanoylcarnitine
63.	C20-DC-Carnitine	Eicosanedioylcarnitine
64.	C22-Carnitine	Behenoylcarnitine
65.	C24&C18:1-DC-Carnitine	Lignoceroylcarnitine
66.	C26-Carnitine	Cerotoylcarnitine

	Metabolites sums or ratios	Observations	Reference
1.	Fischer Ratio	Ratio of branched-chain amino acids to aromatic amino acids - (Ile + Leu + Val) / (Phe + Trp + Tyr)	Ishikawa 2012<br>Dejong 2007
2.	GABR	Global arginine bioavailability ratio - Arg / (Orn + Cit)	Sourij et al. 2011
3.	Glutaminase activity	Glu / Gln ratio	Scalise et al. 2017<br>Mates et al. 2013<br>Erickson et al 2010<br>Lu et al. 2010
4.	Gly Synthesis	Gly / Ser ratio	Wang et al. 2017<br>Zhang et al. 2016
5.	NESS/ESS	Ratio of non- essential to essential amino acids	Stini 1974
6.	Cit Synthesis	Cit / Orn ratio	Choi et al. 2012
7.	NOS activity	Nitric oxide synthase activity - Cit / Arg ratio	Maher et al. 2017
8.	AA Sum	Sum of all amino acids	Tochikubo et al. 2016
9.	Aromatic AA sum	Sum of all aromatic amino acids	Chen et al. 2016<br>Wuertz et al. 2013
10.	BCAA sum	Sum of branched-chain amino acids	Neinast et al. 2019<br>Anzai et al. 2017<br>Allam-Ndoul et al. 2015
11.	ESS AA sum	Sum of all essential amino acids	Tassari et al. 2016
12.	NESS AA sum	Sum of all non-essential amino acids	Hou et al. 2015
13.	Glucogenic AA sum	Sum of glucogenic amino acids	Nuttall et al. 2008
14.	Ketogenic AA sum	Sum of ketogenic amino acids	Puchalska et al. 2017
15.	MetHis Synthesis	Methylhistidine synthesis (MetHis/His ratio)	Kochlik et al. 2018
16.	Orn Synthesis	Ornithine synthesis - Orn / Arg	Caldwell et al. 2015
17.	Valinemia	Indicator of Valinemia and MSUD - Val / Phe	
18.	AC Sum	Sum of all acylcarnitines	Houten et al. 2016
19.	Beta Oxydation	(C2 + C3) / C0	Ottas et al. 2017
20.	C2/C0	Indicator beta oxidation rate	Houten et al. 2016
21.	Pro/Cit	Ratio of Pro to Cit	Moon et al. 2017
22.	Leu/Ala	Indicator of MSUD	
23.	Leu/Phe	Indicator of MSUD	
24.	Phe/Tyr	Indicator of PKU	
25.	C3/C16	Indicator of PA/ Indicator of MA	
26.	C3/C2	Indicator of PA/ Indicator of MA	
27.	C3DC/C10	Indicator of MAL	
28.	C4/C2	Indicator of IBG/ Indicator of SCAD	
29.	C4/C3	Indicator of IBG/ Indicator of SCAD	
30.	C5/C0	Indicator of IVA	
31.	C5/C2	Indicator of IVA	
32.	C5/C3	Indicator of IVA	
33.	C5-OH/C0	Indicator of 3-MCC	
34.	C5-OH/C8	Indicator of 3-MCC	
35.	C5DC/C16	Indicator of GA-1	
36.	C5DC/C5-OH	Indicator of GA-1	
37.	C5DC/C8	Indicator of GA-1	
38.	C8/C10	Indicator of MCAD/ Indicator of MCKAT	

	Metabolites sums or ratios	Observations	Reference
39.	C8/C2	Indicator of MCAD/ Indicator of MCKAT	
40.	C14:1/C12:1	Indicator of VLCAD	
41.	C14:1/C16	Indicator of VLCAD	
42.	C14:1/C2	Indicator of VLCAD	
43.	C14:1/C4	Indicator of VLCAD	
44.	C16-OH/C16	Indicator of TFP/ Indicator of LCHAD	
45.	C24/C22	Indicator of X-ALD/ peroxisomal disorders	
46.	C26/C22	Indicator of X-ALD/ peroxisomal disorders	
